# Vaginal Adsorbent Gel as a Therapeutic Agent: Is a New Era Beginning for HPV?

**DOI:** 10.3390/jcm14144826

**Published:** 2025-07-08

**Authors:** Fatma Ozmen, Sule Gul Aydin, Sevtap Seyfettinoglu, Sevda Bas, Mehmet Ali Narin

**Affiliations:** 1 Department of Gynecologic Oncology, Training and Research Hospital, Ordu University School of Medicine, Ordu 52000, Turkey; 2 Department of Gynecologic Oncology, Malatya Training and Research Hospital, Malatya 44330, Turkey; sulegulaydin@gmail.com; 3 Department of Gynecologic Oncology, Adana City Training and Research Hospital, Health Sciences University School of Medicine, Adana 01230, Turkey; sevtaponcul@gmail.com (S.S.); drsevdabas@gmail.com (S.B.); mali_narin@yahoo.com (M.A.N.)

**Keywords:** HPV, non-surgical treatment, silica, sodium selenite

## Abstract

**Objectives:** Persistent Human Papillomavirus (HPV) infection in the cervix and the preinvasive lesions it causes are significant risk factors for cervical cancer. Therefore, a treatment strategy is necessary to facilitate the clearance of HPV and prevent the progression of preinvasive lesions without causing cervical tissue destruction. This study aimed to evaluate the effectiveness of a vaginal adsorbent gel composed of a hydroxyethyl cellulose matrix formulation containing dispersed silicon dioxide, antioxidant sodium selenite, deflamin, and citric acid in patients with HPV infection. **Methods:** The study was designed as a retrospective cohort study and involved 449 women infected with HPV. For the purposes of the study, the patients were divided into two groups: the treatment group (TG) comprised 207 patients who used the vaginal gel daily for a period of three months, while the control group (CG), consisting of 242 patients, received no treatment under an “active surveillance” protocol. The study’s endpoints encompassed the domains of cytology, histology, and HPV clearance. **Results:** The regression rate of smear pathologies was 24.8% in the control group and 29.0% in the group using the vaginal adsorbent gel. In the first year, the histological regression rate in cervical biopsies was 49.3% in the treatment group and 19.4% in the control group, with a significant difference between groups (*p* < 0.001). Moreover, the clearance rate of HPV types was found to be significantly higher in the group using the vaginal adsorbent gel. **Conclusions**: The findings of this study suggest that the outpatient treatment approach can effectively prevent the oncogenic progression of cervical dysplasia. This alternative method has been shown to be efficacious in preventing the progression of cervical dysplasia and promoting regression. Furthermore, the efficacy of this gel in eradicating HPV has been demonstrated within a 12-month period.

## 1. Introduction

Cervical preinvasive lesions are a substantial health concern that can be prevented from progressing to cancer by early diagnosis and successful treatment. HPV infection is the most significant factor in their etiology [1]. The risk of developing cervical cancer is elevated when cervical HPV infection progresses to pre-invasive lesions and is not diagnosed and treated. Consequently, it is imperative to implement early diagnosis and intervention [2,3].

Cervical intraepithelial neoplasia (CIN) is classified into CIN1, CIN2, and CIN3 based on the depth of invasion within the epithelium. The LAST (Lower Anogenital Squamous Terminology) terminology, developed by the American College of Pathologists and the American Society for Colposcopy and Cervical Pathology (ASCCP), is a classification system for cervical pathologies that considers both the depth of invasion and the progression of HPV infection. The methodology employed in this study is immunohistochemistry, a technique that involves the use of antibodies to detect specific proteins in tissue samples. The classification of lesions is of paramount importance in determining the most efficacious treatment methods. Two distinct classifications of squamous intraepithelial lesions (SIL) are recognized: low-grade squamous intraepithelial lesions (LSIL) and high-grade squamous intraepithelial lesions (HSIL) [4].

The management of CIN can be categorized into two primary modalities: excisional and ablative treatments. Excisional treatment methods, such as conization or Loop Electrosurgical Excision Procedure (LEEP), involve the surgical removal of part of the cervical canal and stromal tissue. Patients who are not at the treatment threshold are monitored with active surveillance to enable early diagnosis and treatment [5].

Studies have shown that around 54 percent of HPV infections undergo spontaneous remission within the first year [6]. The vaginal microbiome and pH levels significantly influence the response to HPV infections [7].

The use of vaginal adsorbent gel (VAG) has been acknowledged in the literature as a supportive treatment option during the ‘active surveillance’ process. As demonstrated in the guidelines of the Austrian Society of Gynecology and Obstetrics (OEGGG), the administration of this VAG has been shown to slow disease progression, reduce HPV viral load, and strengthen the host’s immune response to support the eradication of HPV from the cervix [8]. The VAG used in prior studies typically contains a hydroxyethyl cellulose matrix formulation that includes dispersed silicon dioxide, sodium selenite with antioxidant properties, deflamin, and citric acid [9]. Deflamin is an oligomeric protein that has been isolated from plants. The potential benefits of this natural substance are attributable to its capacity to inhibit matrix metalloproteinases (MMPs) and exhibit anti-inflammatory properties. Consequently, it may serve as a promising antioxidant in the treatment of cancer and inflammatory diseases associated with elevated MMP-9 activity [10]. The concurrent achievement of antibacterial activity and antioxidant effects is a result of the combination of deflamin, selenium, and citric acid [11]. Silicon dioxide enhances the efficacy of natural antimicrobial agents, while lipid coating improves the distribution of particles in the environment and facilitates drug dissolution, thereby enhancing penetration into tissues [12].

HPV plays a pivotal role in the integration of the host genome, the development of cervical preinvasive lesions, and the progression to cancer [13]. Local inflammation facilitates the integration of HPV DNA into cellular chromatin, while systemic inflammation affects the progression of preinvasive lesions by influencing the relationship between HPV persistence and p16 positivity [14,15].

This study evaluates the efficacy of VAG in patients with HPV infection. We will assess the effectiveness of VAG in these patients, particularly those with different grades of preinvasive cervical lesions. Our objective is to investigate this potential relationship in order to identify alternative strategies for the treatment of HPV infection and associated preinvasive lesions.

## 2. Patients and Methods

This study was conducted in accordance with the Helsinki Declaration and was approved by the Ethics Committee of Adana City Training and Research Hospital, University of Health Sciences (approval no: 94/1650/2021). Ethical principles were meticulously adhered to during the process of collecting and utilizing patient data. All patient information was anonymized to ensure the confidentiality of personal data.

From January 2021 to December 2024, a total of 449 individuals aged between 30 and 65 years were enrolled in the study. All participants were found to have a positive HPV test result, classified as either high-risk or low-risk according to the national cervical cancer screening program. The National Cervical Cancer Screening Program in Turkey utilizes DNA profiling methods (SPF10, PCR-DEIA-LiPA25) and positive high-risk HPV test results (Hybrid Capture II, or HC2) to screen women aged 30 to 65 every five years. This screening process is conducted using the Hybrid Capture 2 (HC2) system (Qiagen, Hilden, Germany). It was observed that even individuals with a low-risk HPV status met the criteria for colposcopy during the initial evaluation. Colposcopy is indicated for patients who have tested positive for HPV types 16 and 18, those who have tested positive for other HPV types in conjunction with abnormal cervical–vaginal cytology (CVC) results, patients with cytology results indicating LSIL or more advanced lesions, patients with suspicious findings during inspection and vaginal examination, and patients with a history of postcoital bleeding.

Patients monitored for HPV positivity at our center undergo CVC testing at six months, followed by a repeat HPV test and colposcopy within the first year. In our center, we use Roche Cobas^®^ 4800 HPV tests to evaluate cervical samples during the first year of HPV testing. These tests use the PCR method, and we use ThinPrep (Hologic Inc., Marlborough, MA, USA) liquid-based cytology. A total of 14 genotypes of hr-HPV DNA, including hr-HPV 16 and 18 and other high-risk genotypes (31, 33, 35, 39, 45, 51, 52, 56, 58, 59, 66, 68), were identified using the Roche Cobas.

Patients with HSIL (CIN2-CIN3) lesions are recommended excisional or ablative treatment, while those with LSIL (CIN1) lesions are followed up with active surveillance. During this process, patients are provided with information about alternative medical topical therapy, and their usage preferences are left to their discretion. At follow-up visits, the topical therapy used by patients and their durations of use are recorded in the system.

In this retrospectively designed study, patient data was accessed through a computer system. To determine whether progression or regression occurred, the following regression classifications under the CVC framework should be considered: cytological regression is classified as HSIL → ASC-H (Atypical squamous cells cannot exclude high-grade squamous intraepithelial lesion) → LSIL → ASC-US (Atypical squamous cells of undetermined significance) → NLIM (Negative for Intraepithelial Lesion or Malignancy), while histological regression is classified as CIN3 → CIN2 → CIN1 → Koilocytosis. The results of the HPV test were categorized as positive or negative.

Patients who were monitored with active surveillance using a daily VAG dose for a duration of three months were included in the study, while those who were followed without the use of additional medical topical therapies during the same period constituted the CG. During the active observation period, 207 patients were found to have used VAG, while 242 patients did not use such a topical therapy. For a period of three months following the initial positive test, TG utilized VAG. The earliest sample obtained after treatment was the CVC, which was collected three months after the conclusion of treatment. HPV DNA and cervical samples were collected nine months after the discontinuation of treatment. No samples were collected during the period of VAG usage.

The colposcopy procedure was performed by four specialist gynecological oncologists. The biological materials collected from the patients were evaluated by three pathologists specializing in gynecological pathology.

Patients who had used alternative treatments, such as vaginal spray, vaginal ovules, or oral supplements, with follow-up intervals shorter than four months or longer than eight months, underwent colposcopic evaluation but did not undergo cervical biopsy or endocervical sampling, and those who received immunosuppressive therapy or chemotherapy during the one-year follow-up period were also excluded from the study. In patients with multiple HPV positivity, each HPV type was considered a distinct strain and included in the study as a separate strain that tested negative.

## 3. Statistics

The study used SPSS 27.0 (IBM Inc., Chicago, IL, USA) for statistical analyses and GraphPad Prism 10 for visualizations. Normality of numerical variables was assessed using the Kolmogorov–Smirnov test, histogram analyses, skewness/kurtosis data and Q-Q plots. Quantitative parameters were expressed as mean ± standard deviation and nominal parameters as frequency and percentage (%). Group variances were examined using Levene’s test. Independent t-tests evaluated the relationship between the quantitative parameters. Confusion matrices assessed the relationship of nominal parameters. Pearson’s chi-squared analysis or Fisher’s exact tests were used for statistical analysis. The Bonferroni correction was applied to the comparisons. A multilayer categorization determined the regression status of the findings, and regression conditions were recorded accordingly. Histograms and heat maps were used to create summaries. The type I error rate (α = 0.05) was set at 5% throughout the study, with *p* < 0.050 considered significant.

## 4. Results

A total of 449 patients were eligible for subgroup analysis; 207 of these had HPV-positive cytology and histology results in the TG, while 242 were in the CG. All patients were informed about VAG and asked to indicate their preferences regarding its use. During this process, patients who used VAG regularly for three months were recorded in the data system. Demographic characteristics are presented in Table 1.

There were no significant differences in age groups; the mean ages were 43.69 ± 8.36 and 42.74 ± 8.56 (*p* = 0.236). The proportion of smokers in the TG (20.77%) was lower than that in the CG (36.78%), and smoking was less prevalent in the TG (*p* < 0.001). In the multivariate regression analysis of smoking status with the control group, a significant association supporting continuity was found; however, this significance disappeared in the multivariate logistic regression analysis. After adjusting for confounding factors, no association was found between continuity and the study groups (Table 2).

We examined patients who tested positive for HPV in the initial test and underwent colposcopy, along with cervical and endocervical sampling, within the first year of follow-up. The findings illustrate the regression rates in cervical and endocervical tissues based on annual histological evaluations. The focus was not on the progression or regression of the disease in individual patients but rather on the total number of cervical intraepithelial lesions between groups. The results, detailed in Table 1, provide valuable information about temporal changes in lesions. All patients underwent ECC during biopsy. The comparison of endocervical sampling results obtained at the initial visit and during the one-year follow-up is presented in Table 1.

Both the CG and TG recommended excisional procedures for patients with cervical biopsy and endocervical canal histology results of CIN2 and CIN3. Thirteen patients in the CG and eight patients in the TG with CIN2 cervical biopsy results refused excisional procedures and opted for active surveillance. The number of patients with untreated CIN2 lesions was similar in both groups. Excisional procedures were performed on 13 patients in the CG and 14 patients in the TG. Surgical margins were recorded as negative in all patients who underwent excisional procedures. No statistically significant difference was observed between the groups in terms of the number of patients who underwent excisional procedures (*p* = 0.536).

At the initial evaluation, reflex CVC test results were available for all patients. The initial cervical sampling results did not show any significant differences between the two groups, and NILM was the most common finding. The most common pathological finding in both groups was ASC-US. A comparison of CVC results obtained at 6 months of follow-up for the monitored patients showed that the TG exhibited significantly more favorable outcomes, as indicated in Table 3.

Further analysis of CVC results at 6 months revealed ASC-H in five patients in the CG. The absence of this finding in the TG was notable. Since HSIL was observed in one patient in each group (CG and TG), colposcopic evaluations were expedited for these patients. No advanced histological pathology was detected in any of the seven patients whose colposcopic evaluations were expedited, and these patients were subsequently placed under close observation.

At the initial presentation, 90 patients (37.19%) in the CG and 90 patients (43.48%) in the TG were HPV16-positive. Additionally, 30 patients (12.4%) in the CG and 28 patients (13.53%) in the TG were HPV18-positive. There was no statistically significant difference between the groups in terms of HPV16 and HPV18 positivity (*p* = 0.175). HPV16 was the most commonly detected type in both groups; HPV31 was the second most common type, and HPV18 was third in the TG. In the control group, the second most common type was classified as other HPV (other), while HPV56 was the third most common. When HPV types were compared, no significant difference was observed between the two groups.

In the six-month CVC results, 22 patients (9.09%) in the CG had LSIL, while 6 patients (2.94%) in the TG had LSIL. Additionally, 5 patients (2.07%) in the CG had ASC-H, while no cases were reported in the TG. This study demonstrated a regression rate of 75.0% in ASC-US patients in the CG, while the regression rate in the TG was calculated to be 82.9%. In the CG, regression was observed in 19 out of 26 patients (73.08%), whereas regression was observed in all 16 patients (100%) in the TG. During the data collection process, the incorporation of histological and cytological results emerged as a pivotal element, underscoring their significance in the overall analysis. The cytological results obtained at baseline and at six months, along with the histological findings from cervical and endocervical canal samples collected at baseline and after one year, have been organized into a table based on the regression rates of the groups (Table 4).

During the one-year follow-up period, patients were monitored with cervical biopsy, endocervical canal sampling, and HPV DNA testing, with all results systematically documented. Statistical analysis showed a significant decrease in cervical biopsy results in the TG (*p* < 0.001). The regression rate was 19.4% in the CG and 49.3% in the TG.

Comparison of initial and one-year HPV tests for HPV16 showed that 16 patients (17.8%) were negative in the CG, while 64 patients (71.1%) were negative in the TG. For HPV18, 7 patients (23.3%) were negative in the CG, while 24 patients (85.7%) were negative in the TG. The percentages of negative results for each HPV strain are presented in the tables. These findings indicate a significant difference in negative conversion rates between the groups (Table 5).

In cases of multiple HPV positivity, separate evaluations revealed a total of 355 HPV strains in the CG and 332 strains in the TG. During the one-year follow-up, 197 positive HPV strains were observed in the CG, with 158 becoming negative. In contrast, 150 positive strains were detected in the TG, and 182 of these became negative. During HPV monitoring, it was noted that while some strains yielded negative results, other HPV types showed persistent positivity. Additionally, the results of the first-year evaluations for each HPV type are summarized in Table 6.

## 5. Discussion

The present study demonstrated a statistically significant increase in the rate of histological regression among patients who used the VAG when comparing the results of cervical biopsy from the initial visit to the one-year follow-up (*p* < 0.001). The HPV infection status was also analyzed at the one-year follow-up, revealing that HPV16 positivity became negative in 64 patients (71.1%) in the cohort that used the VAG.

CVC is a frequently preferred technique for monitoring cervical pathologies due to its accessibility. Biomarkers aid in the diagnosis of cytological pathologies [16]. In a prospective randomized controlled trial conducted in the Czech Republic, patients infected with high-risk HPV types (HPV 16, 18, 31, 33, 35, 39, 45, 51, 52, 56, 58, and 59) were included. Individuals identified by p16/Ki-67 positivity and cytological findings were considered. The results of CVC at three months were compared between VAG users and non-users in active surveillance. A statistically significant improvement was observed in the treatment group [17]. In our study, the use of this alternative treatment was left to patient preference, and control CVC samples were taken three months after VAG use. As the pathology reports did not include the p16/Ki-67 biomarkers, no evaluation was made in this regard. Significant regression was observed in the cytology results, and similar results were obtained. In the present study, the use of VAG by patients commenced following their initial hospital presentation.

**Figure 1:**
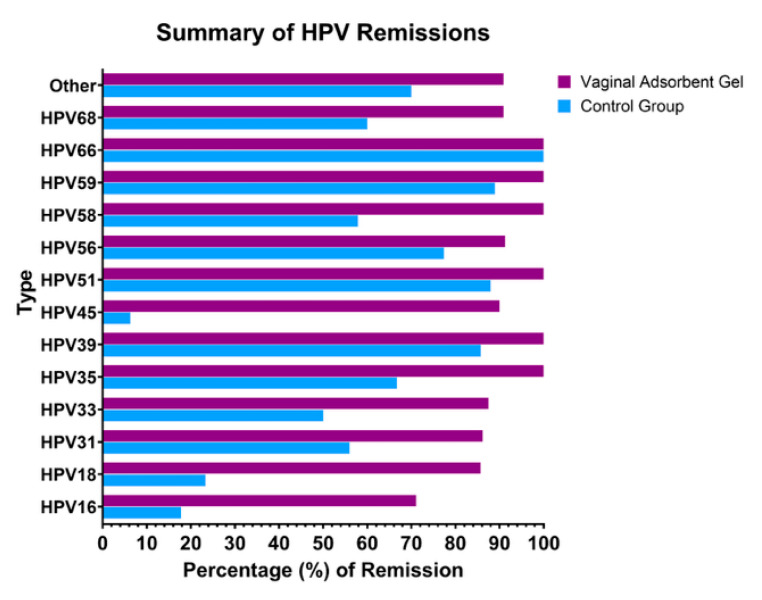
The summary of HPV remissions.

Oncogenic viruses frequently require co-factors to manifest their carcinogenic potential. Oxidative stress has been demonstrated to play a critical role in the carcinogenesis process induced by HPV; for this reason, antioxidant capacity is of significant importance [18,19]. A study published in 2021 evaluated the hypothesis that the antioxidant properties of citric acid and sodium selenite could support HPV eradication by strengthening the vaginal flora. After using a VAG for three months, statistically significant results were obtained in the eradication of high-risk HPV viruses [20]. In this study, regression analysis was performed on high-risk HPV viruses, revealing a total of 14 different HPV types. When subgroups were examined, the clearance rate for HPV16 was 17.8% in the CG and 71.1% in the VAG group. For HPV 18, the negativity rate was 23.3% in the CG, which increased to 85.7% in the VAG group. The negativity rates for this subtype were higher in the VAG group (Figure 1).

The results of this study are consistent with existing literature and support data from other studies indicating higher HPV clearance in the VAG group.

The Population-Based Screening Study Amsterdam (POBASCAM) group conducted a study evaluating viral clearance by HPV type in a large sample and found that HPV16 infections were less likely to be negative at 18 months compared to other high-risk HPV types. Additionally, the HPV types with the lowest clearance rates were identified as HPV16, HPV18, HPV31, and HPV33 [21]. We found that clearance rates for HPV16 and HPV18 were consistent with the POBASCAM data, while clearance rates for HPV31 and HPV33 were higher.

In a prospective randomized study designed to evaluate the effect of VAG on histologic parameters, cervical biopsy results were analyzed in 216 patients diagnosed with p16/Ki67-positive CIN1 and CIN2. In this group of patients, significant regression of CIN lesions was observed in cervical biopsies performed three months after the application of the VAG [22]. Following the treatment period, CVC were collected at the earliest three months later; HPV DNA testing and cervical sampling were conducted nine months after that. Analysis of CVC results shows significant regression in patients using VAG. Histological evaluation of cervical tissue samples obtained nine months after the completion of treatment has revealed confirmed healing (*p* < 0.001). The findings indicate that the effectiveness of VAG, used for a duration of three months, is not limited to short-term results but extends to an efficacy that lasts up to nine months. This finding suggests that the efficacy of the treatment extends beyond the application period, contributing to the prevention of progression over an approximate follow-up duration of nine months. The long-term effects should also be considered in the context of HPV clearance. Furthermore, the treatment has been shown to not only prevent the progression of cervical pathologies but also to reduce the incidence of interventions that may lead to tissue loss or damage in the cervix. These findings support the potential benefits of vaginal application as an alternative therapy for cervical conditions. Future research should be directed toward investigating the long-term effects and underlying mechanisms associated with this vaginal application.

The therapeutic modalities for treating cervical pathologies can be categorized into two primary classifications: excisional and ablative therapies. However, in the context of endocervical canal pathologies, the use of ablative treatment methods is generally not preferred, as these methods lack the capacity to facilitate the evaluation of lesion margins. A review of the literature reveals that there were no studies that histologically evaluated the effects of VAG usage on progression and regression in endocervical canal pathologies. A study was conducted to evaluate the efficacy of topical photodynamic therapy (PDT) employing 5-aminolevulinic acid (5-ALA) in patients diagnosed with endocervical canal pathologies and low-grade squamous intraepithelial lesions (LSIL) due to high-risk HPV infection. The study reported the occurrence of cytological regression and the resolution of HPV infection [23]. In the first year of endocervical sampling, the regression rate was 18.2% in the control group and 15.9% in the group using VAG. The application of agents to the lesion site in endocervical canal pathologies is challenging due to mechanical factors. The use of VAG is unlikely to be effective in patients with endocervical canal pathologies who are under follow-up.

A study of the risk factors for the persistence of HPV indicates that smoking and advanced age are particularly significant variables [24]. The impact of smoking duration and frequency on the persistence of HPV has been extensively documented in the existing literature. Consequently, studies have underscored the imperative for patients to either cease smoking or substantially reduce their usage [25]. The findings of the present study suggest that the prevalence of smoking among patients using VAG is significantly lower. This finding highlights one of the limitations of the study. Following the evaluation of mean age, it was discovered that there were no statistically significant differences between the two groups.

When evaluating the strengths of the study, it is important to note that 449 patients were followed for one year. Despite the single-center design, which could be considered a limitation, the fact that the evaluation was performed by three doctors specializing in gynecology and pathology enhances the overall robustness of the study. Among the limitations, those related to smoking rates are particularly significant. Additionally, it is important to note the retrospective nature of the design. Prospective randomized studies with longer follow-up periods are needed to evaluate the effect of VAG use on cervical pathologies.

The authors declare that they have no conflicts of interest related to this study. The findings, interpretations, and conclusions presented in this manuscript are those of the authors alone and have not been influenced by any external affiliations or financial support. Throughout the study process, the integrity of the research and the impartiality of the analysis have been maintained.

## 6. Conclusions

The utilization of VAG in patients under close surveillance offers a conservative strategy to promote HPV clearance by reducing the progression of cervical preinvasive lesions. Its primary advantage is its suitability for outpatient settings. Additionally, by reducing the progression of cervical intraepithelial lesions, the use of VAG may decrease the number of excisional and ablative treatment methods employed for therapeutic purposes.

## Figures and Tables

**Table 1:** Demographic data, baseline, and one-year cervical biopsy and endocervical curettage results.

		Sample Group Characteristics		
		Control Group (n = 242, 53.9%)	Adsorbent Gel Users (n = 207, 46.1%)	*p*-Value
	Parameter	Description *		
	Age	43.69 ± 8.36	42.74 ± 8.56	0.236 **
	Smoking Status Positive (+)	89 (36.78%)	43 (20.77%)	<0.001 ***
Biopsy	Initial Biopsy			0.009 *
	Koilocytosis	137 (56.61%) ^a^	88 (42.51%) ^b^	
	CIN1	77 (31.82%) ^a^	96 (46.38%) ^b^	
	CIN2	14 (5.79%)	15 (7.25%)	
	CIN3	12 (4.96%)	8 (3.86%)	
	Non-diagnostic	2 (0.83%)	0 (0%)	
	Year 1 Biopsy			<0.001 *
	Koilocytosis	148 (61.16%) ^a^	163 (78.74%) ^b^	
	CIN1	65 (26.86%) ^a^	38 (18.36%) ^b^	
	CIN2	22 (9.09%) ^a^	3 (1.45%) ^b^	
	CIN3	6 (2.48%)	3 (1.45%)	
	Non-diagnostic	1 (0.41%)	0 (0%)	
Endocervical Curettage	Initial Endocervical Curettage			0.799 ***
	Normal Endocervical Tissue	160 (66.12%)	131 (63.29%)	
	CIN1	7 (2.89%)	6 (2.9%)	
	CIN2	0 (0%)	0 (0%)	
	CIN3	1 (0.41%)	1 (0.48%)	
	Endometrial Tissue	7 (2.89%)	8 (3.86%)	
	Mucus	53 (21.9%)	42 (20.29%)	
	Non-diagnostic	14 (5.79%)	19 (9.18%)	
	Year 1 Endocervical Curettage			0.012 *
	Normal Endocervical Tissue	154 (63.64%)	137 (66.18%)	
	CIN1	15 (6.2%) ^a^	2 (0.97%) ^b^	
	CIN2	0 (0%)	0 (0%)	
	CIN3	0 (0%)	1 (0.48%)	
	Endometrial Tissue	2 (0.83%)	3 (1.45%)	
	Mucus	57 (23.55%)	43 (20.77%)	
	Non-diagnostic	14 (5.79%)	21 (10.14%)	

* Fisher’s exact test, ** independent *t*-test, *** Pearson’s chi-square analysis. Items that represent significant differences between columns within the distribution matrix are marked as (^a^) and (^b^). CIN: Cervical intraepithelial neoplasia.

**Table 2:** Univariate and multivariate logistic regression analysis in terms of persistence.

	Univariate LR				Multivariate LR	
Variables	B	Nagelkerke R^2^	OR (95% CI)	*p*	OR	*p*
Age (years)	−0.0002	<0.001	0.999 (0.978–1.022)	0.984	1.002	0.891
Smoking	0.422	0.012	1.525 (1.003–2.319)	0.049	1.432	0.104
Group	0.421	0.014	1.524 (1.046–2.22)	0.028	1.441	0.062

**Table 3:** Distribution analysis of smear characteristics in sample groups at baseline and 6 months.

	Sample Group		
	Control Group (n = 242, 53.9%)	Adsorbent Gel Users (n = 207, 46.1%)	*p*-Value *
Parameter	Distribution *		
Initial cervical–vaginal cytology			0.390
Non-diagnostic	11 (4.55%)	15 (7.25%)	
NILM	150 (61.98%)	119 (57.49%)	
Infection	18 (7.44%)	18 (8.70%)	
ASC-US	32 (%13,22)	35 (%16,91)	
LSIL	26 (10.74%)	16 (7.73%)	
ASC-H	5 (2.07%)	2 (0.97%)	
HSIL	0 (0%)	1 (0.48%)	
AGC	0 (0%)	1 (0.48%)	
6-month cervical–vaginal cytology			0.005
Non-diagnostic	0 (0%)	0 (0%)	
NILM	164 (67.77%)	156 (75.36%)	
Infection	22 (9.09%)	27 (13.04%)	
ASC-US	28 (11.57%)	17 (8.21%)	
LSIL	22 (9.09%) ^a^	6 (2.90%) ^b^	
ASC-H	5 (2.07%) ^a^	0 (0%) ^b^	
HSIL	1 (0.41%)	1 (0.48%)	
AGC	0 (0%)	0 (0%)	

In the distribution matrix, elements that exhibit significant differences between columns are denoted as (^a^) and (^b^). * Fisher’s exact test was applied. NILM: Negative for Intraepithelial Lesion or Malignancy, ASC-US: Atypical squamous cells of undetermined significance, ASC-H: Atypical squamous cells cannot exclude high-grade squamous intraepithelial lesion, HSIL: High-grade squamous intraepithelial lesion, LSIL: low-grade squamous intraepithelial lesions, AGC: Atypical glandular cells.

**Table 4:** Examination of the regression status of findings based on lesions, analyzed separately by groups.

Regression Analysis Of Findings							
Control Group				Adsorbent Gel Users			
	N_B_	N_REG_	Reg (%)		N_B_	N_REG_	Reg (%)
CVC				CVC			
NILM	150	0	0.0%	NILM	119	0	0.0%
Koilocytosis	18	14	77.8%	Koilocytosis	18	12	66.7%
ASC-US	32	24	75.0%	ASC-US	35	29	82.9%
LSIL	26	19	73.1%	LSIL	16	16	100.0%
ASC-H	5	3	60.0%	ASC-H	2	2	100.0%
HSIL	-	-	-	HSIL	1	1	100.0%
Biopsy				Biopsy			
Koilocytosis	137	0	0.0%	Koilocytosis	88	0	0.0%
CIN1	77	28	36.4%	CIN1	96	80	83.3%
CIN2	14	9	64.3%	CIN2	15	15	100.0%
CIN3	12	10	83.3%	CIN3	8	7	87.5%
ECC				ECC			
Mucus	53	0	0.0%	Mucus	42	0	0.0%
Koilocytosis	160	39	24.4%	Koilocytosis	131	27	20.6%
CIN1	7	4	57.1%	CIN1	6	5	83.3%
CIN2	-	-	-	CIN2	-	-	-
CIN3	1	1	100.0%	CIN3	1	1	100.0%

N_B_ = frequency at baseline (n); NREG = number of regressions (n); Reg (%) = percentage of regressions. ASC-H: Atypical squamous cells-cannot exclude high-grade squamous intraepithelial lesion, ASC-US: Atypical squamous cells of undetermined significance, Cin: cervical intraepithelial neoplasia, CVC: cervical–vaginal cytology, ECC: Endocervical curettage, HSIL: high-grade squamous intraepithelial lesion, LSIL: low-grade squamous intraepithelial lesions, NILM: Negative for Intraepithelial Lesion or Malignancy.

**Table 5:** Remission status by HPV types in control group and group using vaginal adsorbent gel.

Number of Cases with Negative Results, N (%) *		
Type	Control Group	Adsorbent Gel Users
HPV16	16 (17.8%)	64 (71.1%)
HPV18	7 (23.3%)	24 (85.7%)
HPV31	14 (56.0%)	25 (86.2%)
HPV33	5 (50.0%)	7 (87.5%)
HPV35	4 (66.7%)	12 (100.0%)
HPV39	12 (85.7%)	15 (100.0%)
HPV45	1 (6.3%)	18 (90.0%)
HPV51	22 (88.0%)	26 (100.0%)
HPV56	24 (77.4%)	21 (91.3%)
HPV58	11 (57.9%)	13 (100.0%)
HPV59	16 (88.9%)	15 (100.0%)
HPV66	1 (100.0%)	3 (100.0%)
HPV68	9 (%60,0)	10 (90.9%)
HPV (Other)	28 (70.0%)	20 (90.9%)

* Cases that initially tested positive and were found to be negative at the 1-year follow-up were considered. HPV: Human papillomavirus.

**Table 6:** Summary of the distribution of HPV tests in the sample at the one-year follow-up.

		**Sample Group**		
		**Control Group** **(n = 242, 53.9%)**	**Adsorbent Gel Users** **(n = 207, 46.1%)**	***p*** **-Value**
**End-of-Year Exam**	**Result**	Distribution *		
**HPV (General) †**	Positive (+)	151 (62.4%)	107 (51.69%)	0.022 *
	Negative (−)	91 (37.6%)	100 (48.31%)	
**HPV Types**				
HPV16	Negative (−)	166 (68.6%)	156 (75.3%)	0.112 *
	Positive (+)	76 (31.4%)	51 (24.64%)	
HPV18	Negative (−)	218 (90.08%)	189 (91.3%)	0.658 *
	Positive (+)	24 (9.92%)	18 (8.7%)	
HPV 31	Negative (−)	230 (95.04%)	197 (95.17%)	0.950 *
	Positive (+)	12 (4.96%)	10 (4.83%)	
HPV68	Negative (−)	232 (95.87%)	205 (99.03%)	0.038 *
	Positive (+)	10 (4.13%)	2 (0.97%)	
HPV56	Negative (−)	235 (97.11%)	200 (96.62%)	0.766*
	Positive (+)	7 (2.89%)	7 (3.38%)	
HPV35	Negative (−)	240 (99.17%)	204 (98.55%)	0.666 **
	Positive (+)	2 (0.83%)	3 (1.45%)	
HPV59	Negative (−)	240 (99.17%)	202 (97.58%)	0.256 **
	Positive (+)	2 (0.83%)	5 (2.42%)	
HPV45	Negative (−)	225 (92.98%)	198 (95.65%)	0.226*
	Positive (+)	17 (7.02%)	9 (4.35%)	
HPV39	Negative (−)	240 (99.17%)	204 (98.55%)	0.666 **
	Positive (+)	2 (0.83%)	3 (1.45%)	
HPV51	Negative (−)	238 (98.35%)	197 (95.17%)	0.053*
	Positive (+)	4 (1.65%)	10 (4.83%)	
HPV58	Negative (−)	234 (96.69%)	205 (99.03%)	0.116 **
	Positive (+)	8 (3.31%)	2 (0.97%)	
HPV33	Negative (−)	236 (97.52%)	204 (98.55%)	0.515 **
	Positive (+)	6 (2.48%)	3 (1.45%)	
HPV52	Negative (−)	234 (96.69%)	200 (96.62%)	0.964*
	Positive (+)	8 (3.31%)	7 (3.38%)	
HPV61	Negative (−)	242 (100%)	206 (99.52%)	0.461 **
	Positive (+)	0 (0%)	1 (0.48%)	
HPV66	Negative (−)	242 (100%)	206 (99.52%)	0.461 **
	Positive (+)	0 (0%)	1 (0.48%)	
HPV69	Negative (−)	242 (100%)	206 (99.52%)	0.461 **
	Positive (+)	0 (%0)	1 (%0,48)	
HPV (other)	Negative (−)	223 (92.15%)	190 (91.79%)	0.888 *
	Pozitif (+)	19 (7.85%)	17 (8.21%)	

* Pearson chi-square analysis. ** Fisher’s exact test. HPV: Human papillomavirus. †: The data represent the combined evaluation of all HPV types.

## Data Availability

The data generated in the present study may be requested from the corresponding author.

## Abbreviations

5-ALA	5-aminolevulinic acid
ASC-H	Atypical squamous cells-cannot exclude high-grade squamous intraepithelial lesion
ASC-US	Atypical squamous cells of undetermined significance
ASCCP	American Society for Colposcopy and Cervical Pathology
CG	control group
CIN	cervical intraepithelial neoplasia
CVC	cervical–vaginal cytology
ECC	endocervical curettage
HPV	Human papillomavirus
HSIL	high-grade squamous intraepithelial lesion
LAST	Lower Anogenital Squamous Terminology
LEEP	Loop Electrosurgical Excision Procedure
LSIL	low-grade squamous intraepithelial lesions
NILM	Negative for Intraepithelial Lesion or Malignancy
OEGGG	Austrian Society of Gynecology and Obstetrics
PDT	topical photodynamic therapy
POBASCAM	Population-Based Screening Study Amsterdam
SIL	squamous intraepithelial lesion
TG	treatment group
VAG	vaginal adsorbent gel
